# Laparoscopic Allograft Spacer Placement to Minimize Bowel Dose During Re-irradiation with Interstitial Brachytherapy

**DOI:** 10.7759/cureus.5958

**Published:** 2019-10-22

**Authors:** Shraddha Dalwadi, Anuj Suri, Aparna Kamat, E. Brian Butler, Andrew M Farach

**Affiliations:** 1 Radiation Oncology, Baylor College of Medicine, Houston, USA; 2 Obstetrics and Gynecology, Houston Methodist Hospital, Houston, USA; 3 Radiation Oncology, Houston Methodist Hospital, Houston, USA

**Keywords:** brachytherapy

## Abstract

In primary or re-irradiation of gynecologic malignancies, achieving optimal dosimetry with adjacent normal tissue becomes challenging. Surgical spacers are tissue-equivalent materials placed within the patient to protect organs at risk from long-term radiation effects and are commonly used in prostate cancer. We report the use of an allograft mesh to protect adhesed bowel from high-dose radiation for definitive treatment of recurrent endometrial cancer.

An 88-year-old female was diagnosed with International Federation of Gynecology and Obstetrics (FIGO) stage II endometrial cancer after she developed urinary frequency, hesitancy, and hematuria. She underwent neoadjuvant chemoradiation, followed by laparoscopic hysterectomy with bilateral salpingo-oophorectomy and adjuvant vaginal cuff brachytherapy. She developed 1.8 cm bilateral vaginal cuff recurrence and was dispositioned for interstitial brachytherapy. An allograft mesh spacer was placed laparoscopically before repeat, high dose rate brachytherapy to protect nearby structures. Dose-escalation was achieved without compromising normal tissue constraints. The patient tolerated the procedure without evidence of long-term toxicity at one year.

Multidisciplinary discussion may help identify patients who would benefit from spacer placement before select dose-escalated radiation therapy. Laparoscopic allograft mesh is one of many types of surgical spacers available for such patients.

## Introduction

With high dose per fraction brachytherapy or stereotactic body radiation therapy, protecting adjacent normal tissue from high dose radiation is challenging and can be dose-limiting. Implanted spacing devices have been utilized to overcome normal tissue constraints and allow for dose-escalation. The first reported use of spacers in radiation therapy was in 1984, when a synthetic pelvic spacer was utilized to spare intestinal loops in the treatment of abdominal malignancies. In the same year, an oral spacer was used to minimize the risk of osteonecrosis [1,2]. Since then, many studies have reported the use of spacers in various anatomical sites. A variety of materials for surgical spacers exist, such as silicone or acrylic resin, blood patch, balloon, collagen, hydrogel, hyaluronic acid, saline, and acellular human dermis [3-5].

Currently, no guidelines exist for the use of spacers in radiotherapy. Reports in the literature describe the use of these techniques to minimize normal tissue dose in head and neck, cervical, and prostate cancer [6-8]. While short-term follow-up shows favorable safety profiles, long-term data is not presently available to evaluate the delayed complications of prophylactic spacer use in brachytherapy. However, in select cases where dose delivery to tumor remains suboptimal without compromising normal structure limits, surgical implants may decrease the risk of post-procedure morbidity without conceding oncologic outcome.

We present an elderly patient with a solitary vaginal cuff recurrence after previous chemoradiation. Acellular dermal matrix spacer was successfully placed laparoscopically at the time of interstitial high-dose rate brachytherapy to minimize bladder and small bowel dose.

## Case presentation

An 88-year-old female was diagnosed with International Federation of Gynecology and Obstetrics (FIGO) stage II endometrial cancer after she developed mixed urinary frequency and hesitancy with hematuria. Performance status at diagnosis was Eastern Cooperative Oncology Group (ECOG) 1. A cervical mass was identified on speculum exam. Biopsy confirmed poorly-differentiated adenocarcinoma of cervical versus endometrial origin. Histopathologic analysis showed negative carcinoembryonic antigen, focally positive vimentin, patchy p16, and negative synaptophysin staining, favoring endometrial primary despite negative estrogen receptor staining. Staging computerized tomography (CT) was negative for metastatic disease. Given the bulk of disease in the cervix and lower uterine segment, neoadjuvant chemoradiation was favored for downstaging. Simulation was performed supine with full and empty bladder for adequate target coverage with motion. Subsequently, planning followed using three-dimensional conformal technique. She began weekly cisplatin and pelvis radiation to 45 Gy. However, treatment course was complicated by intractable diarrhea and subsequent dehydration requiring multiple hospitalizations. Treatment was ultimately suspended after four cycles and 30.6 Gy.

Despite an interrupted course, magnetic resonance imaging (MRI) demonstrated a favorable treatment response and laparoscopic total hysterectomy with bilateral salphingo-oopherectomy was performed. Final pathology showed a 5-cm FIGO grade 2 endometrioid endometrial adenocarcinoma with 0.1/0.9 cm of myometrial invasion and no lymphovascular invasion. Margins were negative. Extensive tumor necrosis and treatment effect was present throughout, including in the cervical stroma, consistent with pre-treatment stage II disease.

Post-operatively, she was treated with high-dose rate vaginal cuff brachytherapy (24 Gy in four fractions prescribed to the surface at 0.5 cm depth of the upper 4 cm of the vagina), given her incomplete course of pelvic external beam radiation therapy. She tolerated treatment well without major complication and was followed closely for surveillance.

**Figure 1:**
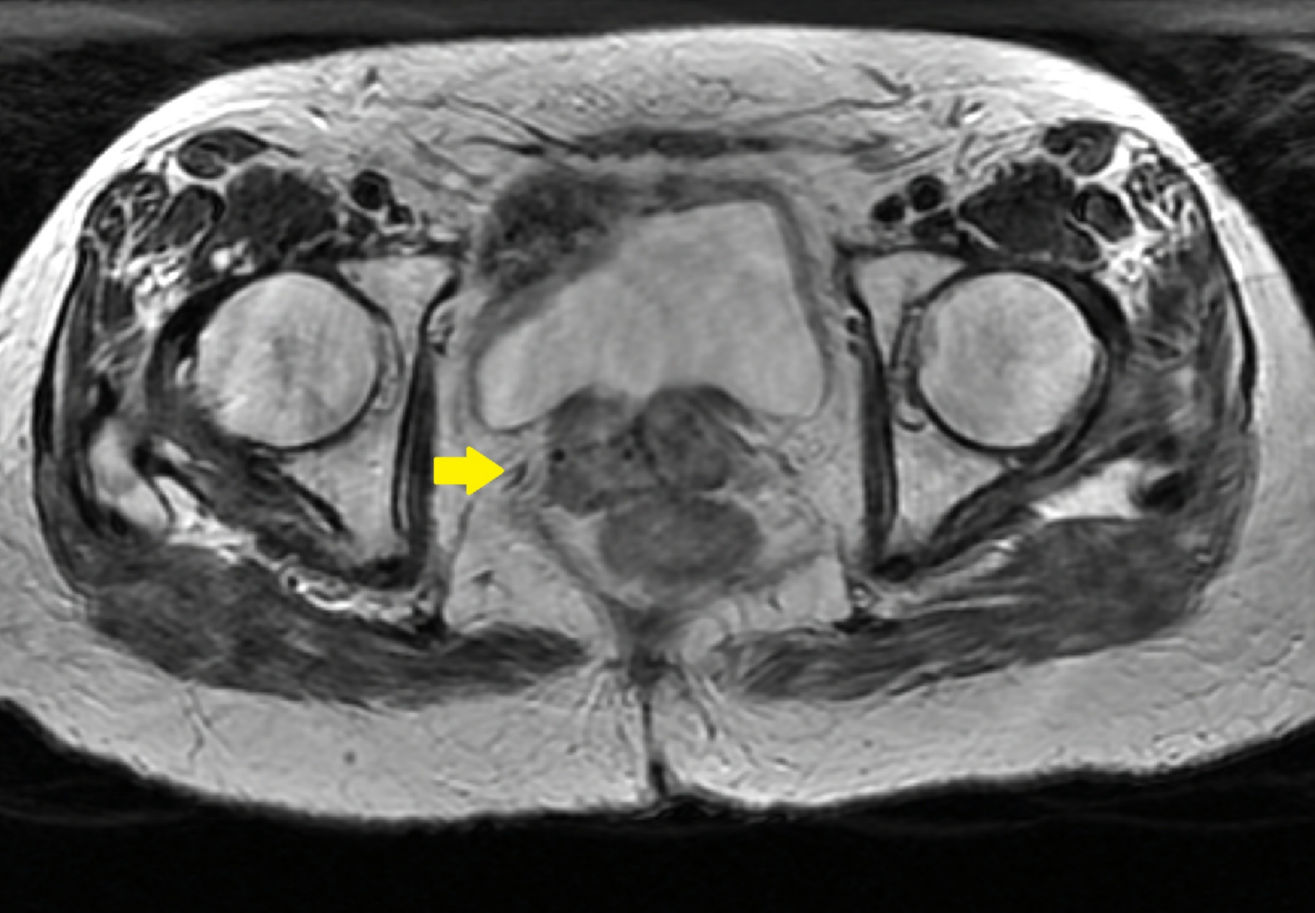
Pre-implant axial magnetic resonance image showing vaginal cuff recurrence

**Figure 2:**
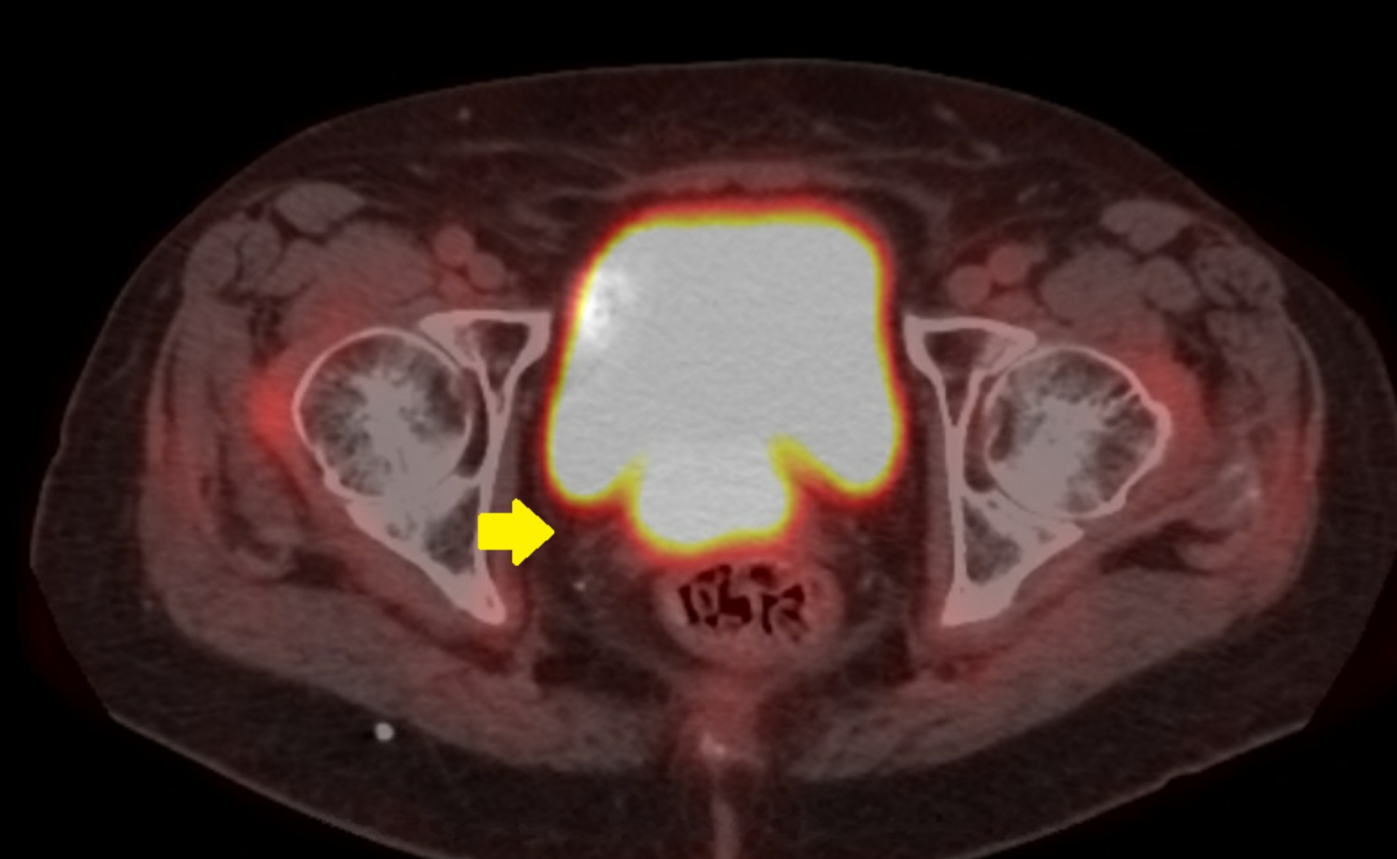
Pre-implant axial positron emission tomography image showing vaginal cuff recurrence

At six months, her speculum exam revealed 2-cm abnormality in the area of the vaginal cuff, within the high-dose brachytherapy field (Figures 1, 2). Biopsy showed recurrent endometrial adenocarcinoma. Positron emission tomography with CT (PET-CT) and pelvic MRI showed no evidence of metastatic disease, only a PET-CT avid 1.8-cm nodular irregularity in the location of the biopsy-proven recurrence. Her case was discussed in a multi-disciplinary setting and the recommendation was made to proceed with local interstitial brachytherapy to allow for coverage of the recurrence and improved dose distribution in comparison to intracavitary techniques.

**Figure 3:**
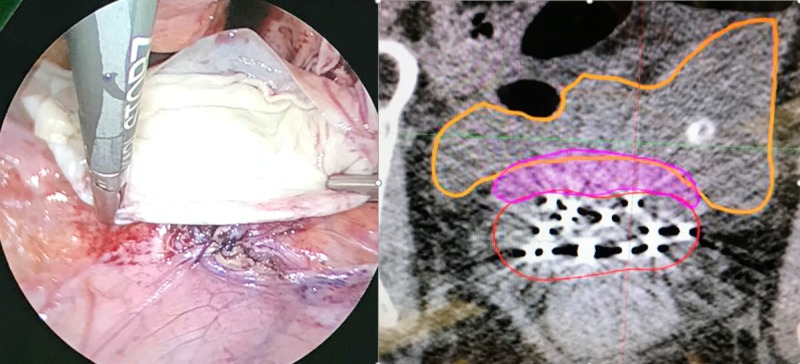
Laparoscopic placement of allograft mesh spacer Biologic mesh (pink) placed between area of recurrence (red) and bladder (orange). Left: intraoperative photo. Right: simulation computerized tomography.

**Figure 4:**
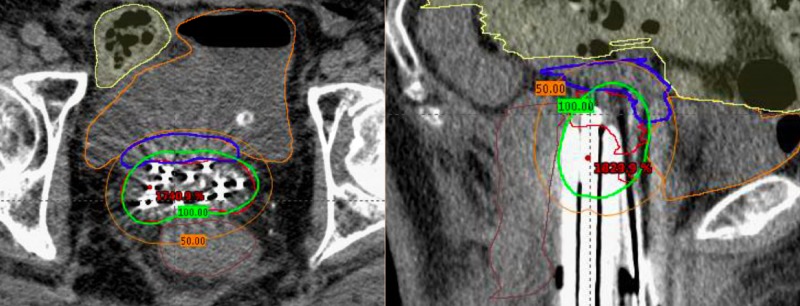
Computerized tomography images of final approved dosimetric plan Left: Axial view. High risk clinical target volume (red) and D100 (green) are separated from the bladder (orange), rectum (brown), and bowel (yellow) by the implanted spacer (blue). Right: Sagittal view.

After a joint discussion between radiation oncology and gynecologic oncology, the decision was made to place allograft mesh spacer on the superior aspect of the vaginal cuff to protect likely adhered bowel given her surgical history and prior pelvic irradiation. She underwent laparoscopy with lysis of adhesions with separation of large bowel from the posterior cul-de-sac and vaginal cuff prior to placement of an dermal matrix spacer (Figure 3). 12 interstitial needles were placed using a Syed-Neblett template intra-operatively during the same case. She underwent CT-based simulation supine with a full bladder. MRI fusion was used to assist in contouring at-risk volumes. The patient completed 30 Gy in five fractions with twice daily fractionation to the area of recurrent disease. Cumulative dose in 2 Gy per day equivalent to bladder, bowel, rectum and sigmoid were limited to their respective tolerances (Figure 4, Table 1) [9-11]. The area of recurrence was escalated to 79.9 Gy, with 49.6 Gy contributed from the Syed implant. The clinical target volume (CTV) was limited by rectal dose, which received a total of 70.8 Gy maximum dose.

**Table 1:** Cumulative dose calculations in 2 Gy per fraction equivalents HDR: High dose rate; EBRT: External beam radiation therapy; CTV: Clinical target volume.

	Dose (Gy)					
	Rectum	Bladder	Sigmoid	Bowel	CTV	
HDR Cylinder	16.3	11.8	2.6	6.2	-
HDR Syed	25.2	32.3	10.1	11.3	49.6
EBRT	29.4	29.4	29.4	29.4	30.1
Total Equivalent Dose	70.8	73.5	42.1	46.9	79.7
Recc Limit	<75	<90	<75	<75	>85

**Figure 5:**
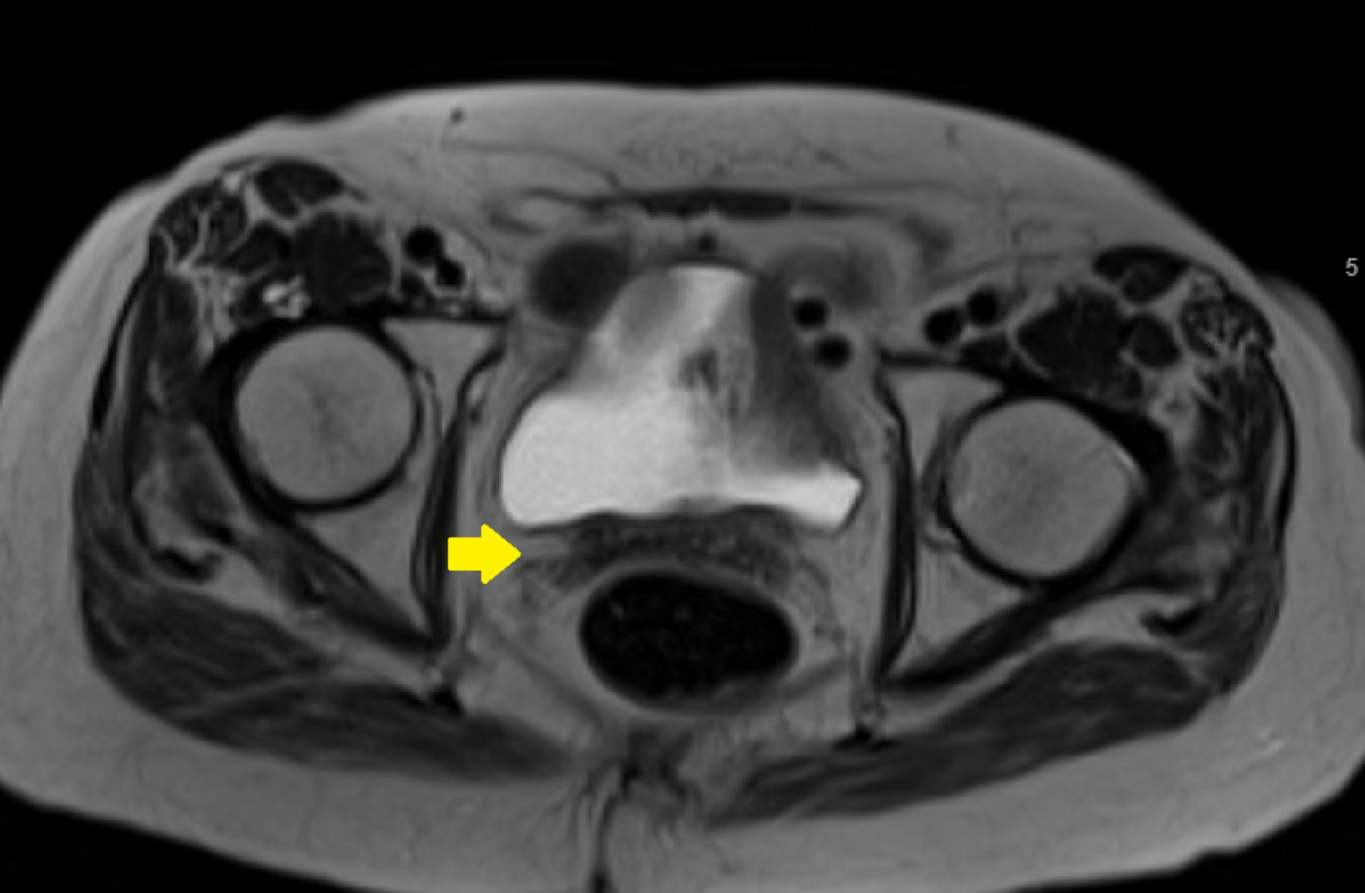
One-month post-treatment axial T2 magnetic resonance image

**Figure 6:**
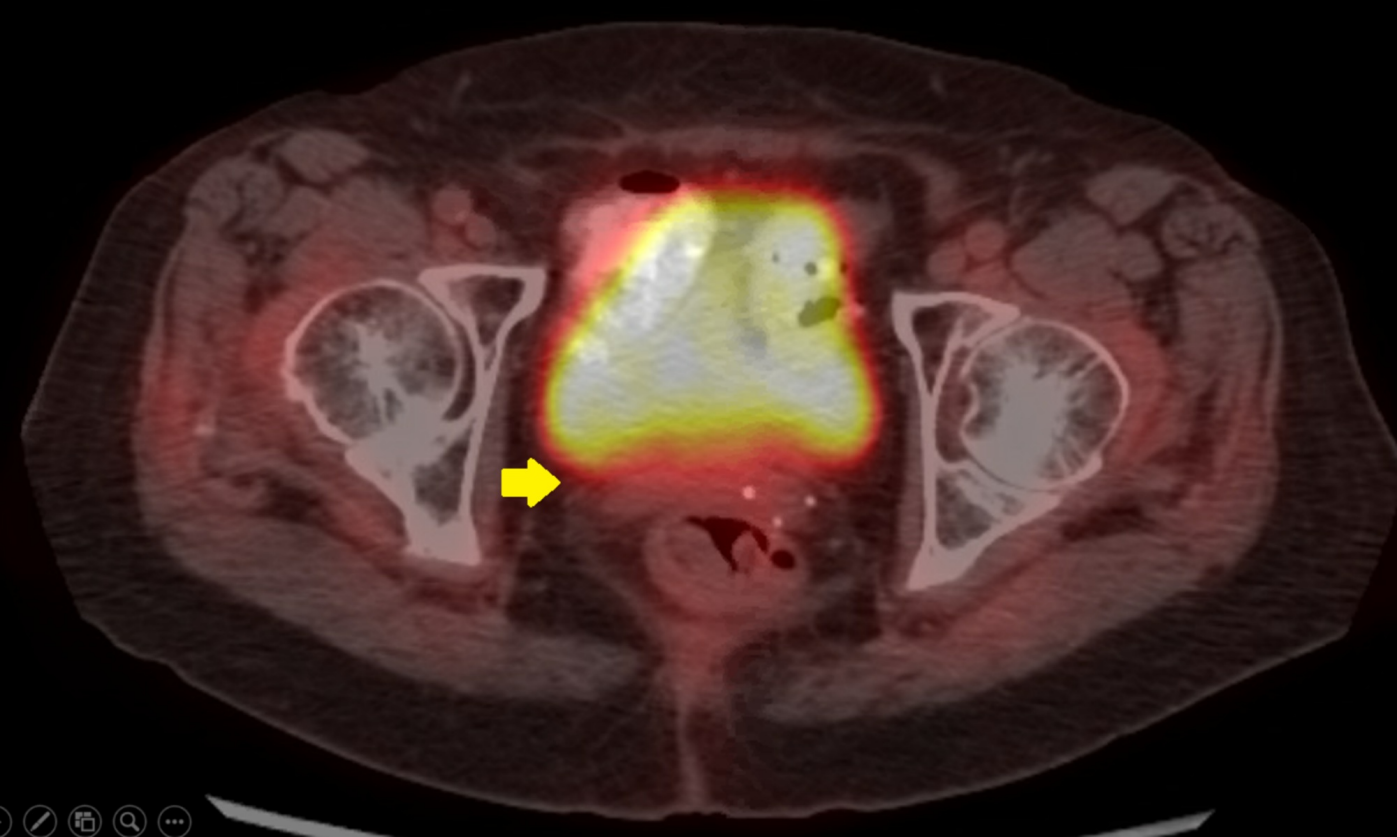
One-month post-Syed salvage brachytherapy axial positron emission tomography image

At one-month follow-up, she reported mild but improving dysuria and vaginal soreness with no radiographic evidence of disease (Figures 5, 6). She otherwise denied symptoms of acute toxicity, including diarrhea. Restaging MRI of the pelvis and PET-CT at 12 months demonstrated a complete clinical response.

## Discussion

We reported a patient with a solitary vaginal cuff recurrence after previous pelvic radiation successfully treated with re-irradiation using interstitial brachytherapy. Dose optimization was facilitated by an allograft mesh spacer to protect the overlying small bowel and bladder. The need for a spacer in our case was discussed due to the patient’s history of previous irradiation and surgery. Allograft mesh is a common material used in general and gynecologic surgery for tissue reinforcement; it was chosen due to its desirable safety profile and relatively low-risk of infection. Laparoscopic technique was utilized given this patient’s concurrent need for lysis of adhesions at the time of placement.

Since the first reported radioprotective spacer use in radiation therapy, multiple types have been developed in attempt to minimize high dose to normal tissue, reduce the risk of short- and long-term treatment toxicity, and overall cost of patient care. While no clear standard guidelines or indications exist in the literature for the use of spacers, a multidisciplinary discussion with review of imaging would be helpful in selecting appropriate patients prior to radiation therapy. Patients receiving brachytherapy or stereotactic body radiotherapy with critical structures adjacent to tumor would likely benefit most, especially in the setting of re-irradiation.

Spacers can be placed open, laparoscopically, endoscopically, or via injection. Minimally invasive injection can be an option if the anatomic location is amenable. Specialty surgery, interventional radiology, and/or procedural medicine consult may be of assistance as available procedures vary by institution. Approach and need may also be determined by the individual’s pre-operative clearance as cancer patients may be too sick to withstand open procedure for a prophylactic benefit to morbidity. Lastly, placement of a surgical implant could be considered at the time of definitive surgery if adjuvant radiation is planned.

Although studies throughout the literature report that spacers are generally safe, patients should be aware of the possible risks associated with the prophylactic placement of a radioprotective foreign material [12-20]. Complication risk is related to technique, anatomic site, and the device itself. The implantation of a spacer subjects patients to procedural risks (bleeding, pain, infection, and iatrogenic trauma to nearby structures) in addition to those associated with anesthesia. Local erosion and allergic reaction is possible. Future radiographic interpretation may be confounded by placement of a surgical spacer. Furthermore, tumor dissemination is a theoretical risk associated with manipulation of tissue in proximity with malignancy. However, no deaths have been reported with implanted or injected spacer for radiation therapy. Ultimately, the benefit of achieving ideal dosimetry and decreasing treatment-related morbidity in these patients may outweigh the small risk of an adverse event.

## Conclusions

It can be challenging to achieve optimal dosimetry in the context of re-irradiation in a postoperative patient. In our case, laparoscopically placed allograft mesh spacer was used prior to interstitial brachytherapy to achieve definitive doses to known recurrence while sparing normal tissue.
